# Microarray analysis of ncRNA expression patterns in *Caenorhabditis elegans *after RNAi against snoRNA associated proteins

**DOI:** 10.1186/1471-2164-9-278

**Published:** 2008-06-11

**Authors:** Muhammad Nauman Aftab, Housheng He, Geir Skogerbø, Runsheng Chen

**Affiliations:** 1 Bioinformatics Laboratory and National Laboratory of Biomacromolecules, Institute of Biophysics, Chinese Academy of Sciences Beijing 100101, PR China; 2 Bioinformatics Research Group, Key Laboratory of Intelligent Information Processing, Institute of Computing Technology, Chinese Academy of Science, Beijing 100080, PR China; 3 Chinese National Human Genome Center, Beijing 100176, PR China; 4 Graduate School of the Chinese Academy of Science, Beijing 100080, PR China

## Abstract

**Background:**

Short non-coding RNAs (ncRNAs) perform their cellular functions in ribonucleoprotein (RNP) complexes, which are also essential for maintaining the stability of the ncRNAs. Depletion of individual protein components of non-coding ribonucleoprotein (ncRNP) particles by RNA interference (RNAi) may therefore affect expression levels of the corresponding ncRNA, and depletion of candidate associated proteins may constitute an alternative strategy when investigating ncRNA-protein interactions and ncRNA functions. Therefore, we carried out a pilot study in which the effects of RNAi against protein components of small nucleolar RNPs (snoRNPs) in *Caenorhabditis elegans *were observed on an ncRNA microarray.

**Results:**

RNAi against individual *C. elegans *protein components of snoRNPs produced strongly reduced mRNA levels and distinct phenotypes for all targeted proteins. For each type of snoRNP, individual depletion of at least three of the four protein components produced significant (P ≦ 1.2 × 10^-5^) reductions in the expression levels of the corresponding small nucleolar RNAs (snoRNAs), whereas the expression levels of other ncRNAs were largely unaffected. The effects of depletion of individual proteins were in accordance with snoRNP structure analyses obtained in other species for all but two of the eight targeted proteins. Variations in snoRNA size, sequence and secondary structure characteristics were not systematically reflected in the affinity for individual protein component of snoRNPs. The data supported the classification of nearly all annotated snoRNAs and suggested the presence of several novel snoRNAs among unclassified short ncRNA transcripts. A number of transcripts containing canonical Sm binding element sequences (Sm Y RNAs) also showed reduced expression after depletion of protein components of C/D box snoRNPs, whereas the expression of some stem-bulge RNAs (sbRNAs) was increased after depletion of the same proteins.

**Conclusion:**

The study confirms observations made for other organisms, where reduced ncRNA levels after depletion of protein components of ncRNPs were noted, and shows that such reductions in expression levels occur across entire sets of ncRNA. Thereby, the study also demonstrates the feasibility of combining RNAi against candidate proteins with ncRNA microarray analysis to investigate ncRNA-protein interactions and hence ncRNA cellular functions.

## Background

Short (50–500 nucleotides (nt)) non-coding RNAs (ncRNAs) generally appear to function through protein complexes. A number of such complexes are known to operate in various essential cellular functions (e.g. the spliceosome, SRP, snoRNPs, 7SK-TEFb [1] and others). Short ncRNAs of unknown function are being detected in increasing numbers in a variety of organisms [2-4]. Cloning of *C. elegans *short ncRNAs identified 100 novel transcripts of which 31% could not be ascribed to any previously known class of RNAs [5], while a recent tiling microarray analysis indicated the existence of approximately 1200 additional short transcripts for which sequence and secondary structure analysis revealed few clues to their cellular functions [6].

RNA interference (RNAi) is a post-transcriptional sequence-specific gene silencing mechanism whose practical application was first demonstrated in *C. elegans *[7]. Tests in our lab (data not shown) suggest that *C. elegans *short ncRNAs are recalcitrant to knock-down by RNAi, irrespective of ncRNA class or mode of RNAi application, thereby rendering unavailable a potentially efficient tool for functional analysis of such transcripts. It was observed, though, that RNAi depletion of individual protein components of ncRNA complexes in a number of cases would also substantially reduce the expression levels of the associated ncRNAs. Such effects have been reported for small nucleolar RNAs (snoRNAs) in yeast [8-12], and a similar approach has been used to analyse the potential involvement of telomerase RNA in human cancers [13]. As the majority of known short ncRNAs appear to act through ribonucleoprotein complexes, we hypothesised that this effect could be employed to identify novel ncRNA-protein interactions which in turn might lead towards elucidating ncRNA functions. If proteins belonging to specific complexes or pathways are depleted by RNAi, which in *C. elegans *can be easily achieved by feeding worms with *E. coli *expressing double-stranded RNA (dsRNA) corresponding to fragments of the targeted protein gene, effects on the ncRNA population can be observed with a specifically designed microarray, and ncRNA-protein interactions subsequently inferred from the reduced expression levels of associated ncRNAs. To test the feasibility of this approach, we depleted each protein associated with both classes of snoRNAs, and observed the effect with a microarray [14] containing probes against 137 *C. elegans *short ncRNAs (tRNAs excluded). Approximately 70 snoRNAs are known in this organism, but very few studies have specifically addressed *C. elegans *snoRNAs. Therefore, in addition to serving as a pilot project for the possible analysis of the full short ncRNA complement in *C. elegans *(estimated to include more than a thousand species [6]) through RNAi knock-down of candidate protein components of ncRNPs, the study should also provide further details on the worm snoRNA-protein relationship. Furthermore, the study will contribute to correct annotation of transcripts with indistinct snoRNA characteristics, an issue that was raised in a recent study [15].

Small nucleolar RNAs are a class of ncRNAs that function in site-specific 2'-O-ribose methylation (C/D box snoRNAs [16,17]) and pseudouridylation (H/ACA box snoRNAs [17]) of rRNAs. The two snoRNA subclasses associate with specific sets of proteins to form snoRNPs, each of which consists of a C/D box or a H/ACA box guide RNA and four associated C/D box or H/ACA box snoRNP proteins. The H/ACA box snoRNAs are composed of two hairpins of varying length separated by short single-strand regions [17-19], and associate with four different evolutionary conserved core proteins, the pseudouridylase Cbf5 (NAP57 in human and mouse), NHP2, NOP10 and GAR1 [8-10,20]. The C/D box snoRNAs contain two conserved sequence elements, boxes C (UGAUGA) and D (CUGA), that are positioned at the 5' and 3' ends of the molecules, respectively [20], and are often flanked by short complementary regions that form short double-stranded stem structures, thought to bring the two boxes in closer proximity [21]. The C/D box snoRNPs are also composed of four core proteins, namely the Snu13, NOP56, NOP58 and NOP1 (fibrillarin) [11,22-26].

As additional controls we also used RNAi to knock down protein components of the signal recognition particle (SRP) and the Y RNP, and observed the effect of this on SRP (or 7 SL) RNA and Y RNA respectively, by Northern analysis. The SRP is an RNP complex consisting of SRP RNA and six different proteins [27] involved in directing nascent peptide chains to the endoplasmic reticulum [28,29]. The Y RNA is associated with the Ro60 and La proteins [30-32], and appears to play a role in rRNA quality control [33,34].

## Results and discussion

In order to observe the effects on the ncRNA expression levels, we carried out RNAi against protein components of the corresponding ncRNPs. PCR fragments of the targeted ncRNP protein genes were cloned into the L4440 plasmid, which induces synthesis of the corresponding dsRNA fragments when transformed into *E. coli *HT115 (see Material and Methods). Results of the RNAis performed against protein components of ncRNPs were observed on the *C. elegans *phenotype and through Northern blots and microarray analysis of the expression levels of the corresponding mRNAs and of both cognate and non-cognate ncRNAs.

### Depletion of snoRNA associated proteins produce distinct phenotypes

**Figure 1:**
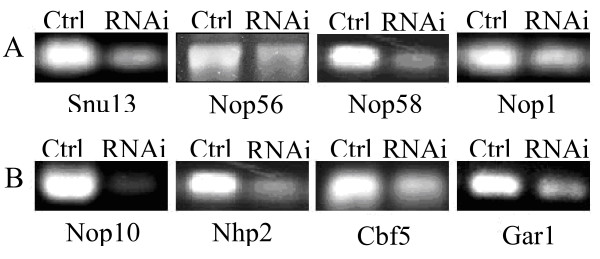
**Northern blots of mRNAs encoding protein components of snoRNPs**. A. C/D box snoRNA-associated protein mRNAs (Snu13, Nop56, Nop58 and Nop1) after depletion by RNAi. B. H/ACA box snoRNA-associated protein mRNAs (Nhp2, Nop10, Cbf5 and Gar1) after depletion by RNAi.

At first we investigated the effects of down-regulation of the C/D box snoRNA proteins, Snu13, Nop56, Nop58 and Nop1. RNAi against each of the four C/D box snoRNP proteins produced distinct phenotypes. Depletion of Nop1, thought to be the methyltransferase [35,36], resulted in a majority of the worms (~60%) showing a sterile phenotype similar to what had been previously reported in worms [37,38]. Depletion of Snu13 resulted in 100% larval lethal worms, also in accordance with former investigations [37,39]. Depletion of Nop56 and Nop58 produced worms with ~80% and ~95% sterile phenotypes, respectively. Most of these sterile worms (~80%) also showed protruding bulges of varying size located at the vulva. When sterile worms with and without protruding bulges were examined under high magnification, it was observed that both types of worms lacked eggs. Previous Nop56 and Nop58 depletion studies in *C. elegans *also resulted in sterile phenotypes and protruding vulvas [37,40]. Depletion of Snu13, Nop56 and Nop58 in yeast also yielded distinct phenotypes [11,26,41]. It is likely that the phenotypes obtained for the down-regulated proteins are the result of the inability to assemble a functional snoRNP complex [42], and not a specific effect of reduced ncRNA expression levels (see below). For each down-regulated C/D box snoRNP gene, total RNA was extracted after 72 hours of feeding. Northern blots were performed to confirm the RNAi depletion of the C/D box snoRNP mRNA genes compared with control worms (Figure 1A).

Similarly, depletion of each of the H/ACA box snoRNP components Nop10, Cbf5, Nhp2 and Gar1 produced distinct phenotypes. In the case of Cbf5, the pseudouridine synthase [9,42-44] of the complex, ~60–70% of the worms were found to be sterile after 72 hours of feeding with dsRNA synthesizing *E. coli*. Previous studies in which Cbf5 was depleted yielded an abnormal phenotype in yeast [9] and a sterile phenotype in *C. elegans *[38], while reduced expression of the human Cbf5 homologue *dyskerin *leads to the condition dyskeratosis congenita [45,46]. Depletion of the Nhp2 protein resulted in 100% of worms with slow growth rates, in agreement with previous studies in *C. elegans *[37,47]. Similarly, RNAi down-regulation of the remaining two genes (Nop10 and Gar1) also produced approximately 95% and 80% sterile worms respectively, after 72 hours of feeding, demonstrating the essential role played by these two proteins [8,48]. Previous Nop10 and Gar1 depletion studies in *C. elegans *also produced a sterile phenotype [38,47]. For each target protein gene (H/ACA box snoRNP mRNA) Northern analysis confirmed a strong reduction in mRNA levels (Figure 1B), and it is reasonable to think that the obtained phenotypes may be a consequence of a loss of function of the targeted protein (or the corresponding RNP complex) rather than an effect of the reduction of any specific ncRNA (see below).

### snoRNP depletions specifically reduces snoRNA expression levels

**Figure 2:**
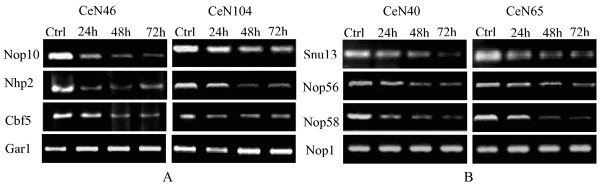
**Effects of RNAi against protein components of snoRNPs on snoRNA expression levels**. A. Northern blots of C/D box snoRNAs CeN40 and CeN65 after RNAi against C/D snoRNP component proteins Snu13, Nop1, Nop58 and Nop56. B. Northern blots of H/ACA box snoRNAs CeN46 and CeN104 after RNAi against H/ACA snoRNP component proteins Nop10, Cbf5, Nhp2 and Gar1.

We then investigated the effect of RNAi against snoRNP mRNAs on the snoRNA expression levels. For this purpose, total RNA was extracted at equal time intervals after depletion of each of the snoRNP component proteins. Northern analyses of four snoRNAs showed that the levels of both C/D box and H/ACA box snoRNAs fell considerably after depletion of several of their respective protein components of snoRNPs (Figure 2). To make sure that such effects are not limited to snoRNAs, we also depleted protein components of the signal recognition particle (SRP) and Y RNP and observed the impact on SRP RNA and Y RNA expression levels, respectively. For both RNPs, depletion of several of the protein components substantially reduced the expression level of the corresponding ncRNA (see Additional file 1).

**Figure 3:**
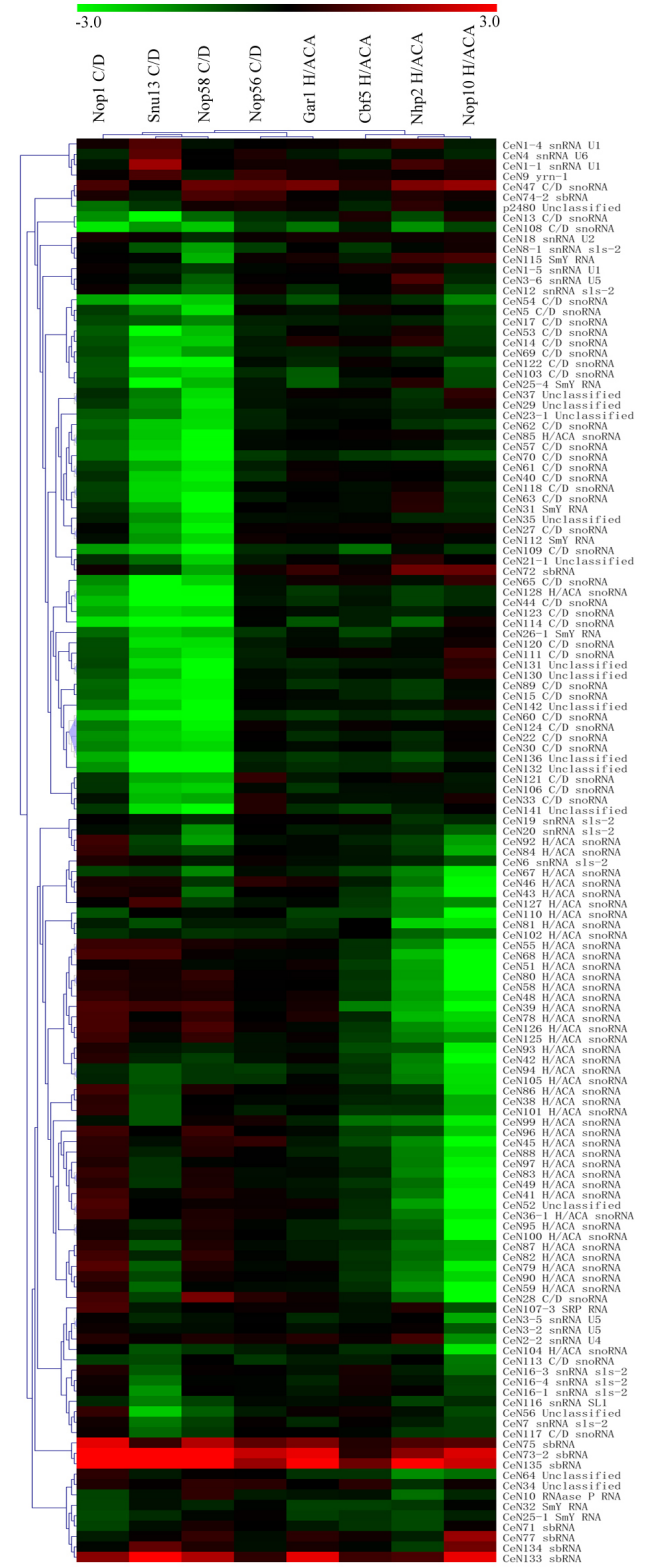
**Microarray expression profiles of all analyzed ncRNAs after individual depletion of all snoRNP proteins**. See Additional file 5 for further details concerning the ncRNA clustering.

Gene expression profiling using microarrays allows for the study of changes in the RNA levels of a large number of genes simultaneously, and we have previously developed a microarray system for profiling the expression of most known *C. elegans *ncRNAs, including snoRNAs [14]. Analysis employing this microarray showed that the levels of both C/D box and H/ACA box snoRNAs fell considerably after depletion of several of the protein components of their respective snoRNPs (Figure 3, Table 1, Additional file  5). The effects on the U snRNAs, SRP RNA and RNase P (assumed not be directly influenced by the depletion of any of the snoRNP components) were negligible (see Additional file 1). As is clearly visible from the microarray data, there were no effects of depletion of protein components of snoRNPs on the opposite type of snoRNA expression levels; i.e. depletion of protein components of C/D box snoRNPs did not affect the expression levels of H/ACA box snoRNAs, and *vice versa *(Figure 3, Additional file 1).

**Table 1:** Average (Av.) relative ncRNA expression levels after RNAi depletion of individual protein components of snoRNPs.

		ncRNA class											
		
Protein depleted		C/D snoRNAs		H/ACA snoRNAs		snRNAs		Sm Y RNAs		sbRNAs		Other ncRNAs^a^	
		
		Av.	P	Av.	P	Av.	P	Av.	P	Av.	P	Av.	P
C/D box snoRNPs	Snu13	0.24	1.6 × 10^-6^	0.83	2.9 × 10^-3^	0.99	1.9 × 10^-2^	0.48	4.4 × 10^-1^	5.44	6.6 × 10^-3^	0.52	4.9 × 10^-1^
	Nop1	0.55	1.2 × 10^-5^	1.31	1.6 × 10^-3^	1.07	5.1 × 10^-2^	0.68	7.7 × 10^-1^	5.70	1.3 × 10^-1^	0.84	4.9 × 10^-1^
	Nop58	0.33	1.4 × 10^-6^	1.03	1.2 × 10^-4^	0.76	1.5 × 10^-2^	0.38	3.5 × 10^-1^	5.39	1.3 × 10^-1^	0.53	3.9 × 10^-2^
	Nop56	0.93	3.7 × 10^-2^	0.96	8.2 × 10^-1^	1.03	2.8 × 10^-1^	0.96	8.3 × 10^-1^	1.98	4.1 × 10^-4^	0.99	8.9 × 10^-1^
	Gar1	0.89	7.3 × 10^-2^	0.93	7.1 × 10^-1^	0.93	8.2 × 10^-1^	0.81	8.8 × 10^-2^	5.61	2.7 × 10^-7^	0.91	9.0 × 10^-1^
H/ACA Box snoRNPs	Cbf-5	0.92	7.8 × 10^-2^	0.68	1.2 × 10^-5^	1.01	9.3 × 10^-3^	0.76	3.6 × 10^-1^	1.28	8.4 × 10^-3^	0.90	3.8 × 10^-1^
	Nhp2	0.88	1.4 × 10^-2^	0.41	7.4 × 10^-9^	1.09	3.0 × 10^-3^	1.07	9.6 × 10^-2^	3.54	6.3 × 10^-3^	0.80	3.7 × 10^-1^
	Nop10	0.87	3.2 × 10^-3^	0.19	1.3 × 10^-8^	0.68	6.0 × 10^-2^	0.97	1.2 × 10^-1^	3.42	5.8 × 10^-3^	0.93	5.7 × 10^-2^

Relative expression was calculated as *treated sample *(RNAi; Cy5)/*control *(Cy3). Statistical significance was calculated using a two tailed student *t*-test.

^a ^"Other ncRNAs" include SRP RNA, RNase P RNA and unclassified ncRNAs.

The data agrees over all with a number of small-scale studies reported in the literatures [8,26]. With regards to the H/ACA box snoRNAs, depletion of Gar1 appears to have no effect on their expression levels (as observed in both Northern and microarray data), despite a distinct phenotype and clearly reduced mRNA levels (Figure 3, Additional file 1). This is in accordance with previous studies [49] and likely reflects the peripheral position of the Gar1 in the H/ACA box snoRNP particle, revealed by structural studies in archaea [50] and eukaryotes [51], and is also true for *C. elegans*. However, consistently negative effects on the H/ACA box snoRNA expression levels after depletion of Nop10 were unexpected. Nop10 knock-down is known to affect H/ACA transcript levels in yeast [8,26]. However, structural studies do not support a very central position in the H/ACA box snoRNP particle for this protein [50,51]. Whereas the effect of Nop10 depletion might be an experimental artefact, the possibility remains for a structural configuration of the nematode H/ACA box snoRNP particle with Nop10 having a more central position than in other studied organisms. RNAi against the larger, pseudouridinylase Cbf5 had a less pronounced (although statistically significant; P = 1.2 × 10^-5^, Table 1) effect on H/ACA box snoRNA levels in the microarray, possibly due to incomplete depletion of this protein.

Depletion of the protein components of C/D box snoRNPs resulted in a marked and consistent reduction in the expression levels of nearly all transcripts annotated as C/D box snoRNAs for proteins Snu13 and Nop58 (Figure 3, Additional file 1). These results are similar to findings deriving from analogous experiments on Nop58 and Snu13 performed in yeast [11,26]. On the other hand, depletion of Nop56 appears to have no effect on C/D box snoRNA expression levels (as observed in both Northern blots and microarray data), despite a distinct phenotype and a reduction in Nop56 mRNA levels. Nop56 has been reported to be dispensable for C/D box snoRNA stability in yeast [24] and our result is in accordance with this study. Moreover, the effect of RNAi against Nop1 on the C/D box snoRNA expression levels was slightly weaker (P = 1.2 × 10^-5^, Table 1) than that observed on Snu13 and Nop58, which may be due to incomplete depletion of the protein, as also indicated by the mRNA Northern blot results (Figure 1A) and the lower frequency of worms (60%) with a distinct phenotype.

Despite maintaining certain common sequence and secondary structure elements, snoRNAs are quite variable in size, sequence and structure, and hence one may expect this variation to translate into differential affinities for the different protein components of the snoRNPs, and consequently into differential effects of the individual protein depletions. However, the relative response to depletion of the different proteins were consistent across individual snoRNAs (Figure 3, Additional file 1). We also analysed specific sequence and structural features of the individual snoRNAs, but found no systematic variable that might explain their overall differential reactions to depletions protein components of the snoRNPs.

### snoRNA annotation

The classification of small non-coding transcripts such as snoRNAs, primarily depends on secondary structure characteristics and short sequence motifs, both of which can be quite variable, and therefore classification of a number of ncRNAs has recently been questioned [15]. Specific snoRNA expression reduction after depletion of protein components of the snoRNPs might therefore also be used to support (or oppose) previous classifications of the *C. elegans *small ncRNA complement. Although snoRNA annotations and the microarray data were in sound agreement, two of the 45 transcripts annotated as H/ACA box snoRNAs and four of the 38 annotated C/D box snoRNAs did not cluster with the assumed snoRNA category (Figure 3, Additional file 1). The two unsupported H/ACA box snoRNAs (CeN128 and CeN85) were not only unaffected by depletion of protein components of the H/ACA box snoRNPs, but rather showed reduced expression levels after depletion of protein components of C/D box snoRNPs, supporting a recent claim that one of these (CeN128) belongs to the C/D box snoRNA sub-class [15]. Similarly, among the four unsupported C/D box snoRNAs, two (CeN28 and CeN113) actually clustered with the H/ACA box snoRNAs, whereas the remaining two (CeN47 and CeN117) displayed expression profiles that were untypical of both snoRNA categories.

### Effects on non-snoRNAs

Whereas the expression levels of spliceosomal U snRNAs were generally not influenced by depletion of protein components of the snoRNPs, the expression levels of two other groups of ncRNAs showed more marked effects. One of these groups is Sm Y RNAs [52] (or snlRNAs [5]), of which five (Sm Y RNAs CeN25-4, CeN26-1, CeN31, CeN112 and CeN115) out of the eight transcripts represented on the microarray showed reduced expression levels after the depletion of protein components of the C/D box snoRNPs (see Additional file 1), while generally remaining stable after depletion protein components of the of H/ACA box snoRNPs. This is an unexpected outcome, as Sm Y RNAs are defined by the presence of an Sm protein binding element within their sequences, an element commonly found in spliceosomal snRNAs, and at least one Sm Y RNA has been shown to associate with proteins in the spliceosomal apparatus [52]. The *C. elegans *transcript annotated as C/D box snoRNA U3 [53] also contains an Sm protein binding element and behaves as a typical C/D box snoRNA in the microarray (Figure 3). The possibility that a few (five) of the Sm Y RNA transcripts may have been wrongly annotated and hence may possibly belong to the class of C/D box snoRNAs has been suggested [15]. However, Sm Y RNAs appear to be a rather numerous class, with 14 verified loci [14] and possibly up to 40 putative loci [6]) found thus far, of which only 8 are represented on the present microarray. Although it is still possible that some of these transcripts might have been wrongly annotated, actually being C/D box snoRNAs, their distinct Sm protein binding elements and preserved upstream sequence motifs [6] might lead to the assumption that a number of *C. elegans *non-codingtranscripts may have sequence characteristics common to both snRNAs and snoRNAs. This may reflect some aspect of the complex trans-splicing operations of nematode mRNA and operon processing [6,54].

The other group of ncRNAs with a marked response were the recently identified stem-bulge RNAs (sbRNAs; [5]), of which four transcripts (CeN73-2, CeN75, CeN133 and CeN135) showed strongly increased expression levels after depletion of the protein components of the snoRNPs (Figure 3, Additional file 1). No sbRNA showed reduced expression with the knock down of any of the C/D box and H/ACA box associated proteins (see Additional file 1).

The expression pattern of previously unclassified ncRNAs was also analysed. The microarray data showed that out of 9 unclassified ncRNAs, two transcripts (CeN52 and CeN64) reacted similarly to annotated H/ACA box snoRNAs while another six (CeN56, CeN37, CeN29, CeN35, CeN23-1, CeN21-1) revealed a reduced expression after C/D box snoRNPs depletion (Figure 3, Additional file 1). Secondary structure analysis of these ncRNAs further supports the classification indicated by the microarray data (see Additional file 2). One ncRNA (CeN34) remained stable after depletion of all snoRNPs and it is most likely not functionally related to snoRNAs.

## Conclusion

In conclusion, this study demonstrates that one can feasibly combine RNAi knock-down of known or potential protein components of ncRNPs with microarray analysis to detect ncRNA-protein interactions. *In lieu *of an efficient RNAi methodology for short ncRNAs depletion, this methodology may thus be utilized to elucidate functional properties of the rapidly increasing number of small transcripts identified in whole genome mapping approaches (e.g. tiling microarray studies [6,54]). The study also confirms the observation that removal of protein components of snoRNPs may strongly reduce expression of the corresponding class of snoRNAs also applies in *C. elegans*, and that this reduction in expression is experienced across entire sets of related transcripts. In addition, the analysis indicates that despite considerable variation in size, sequence and secondary structure characteristics commonly found within a given category of transcripts (e.g. C/D box snoRNAs) their affinity for the core proteins component of the ncRNPs is not systematically affected.

## Methods

### Microarray construction

We previously described the design of a microarray for expression analysis of 137 *C. elegans *ncRNAs [14]. Three house-keeping genes were used as positive internal controls, while SSC buffer and oligos with no homology (<9 bp identity) to the *C. elegans *genome were used as negative hybridization controls, and two rice mRNA oligos with 14 bp identity to two *C. elegans *mRNAs as non-specific hybridization controls. Oligos were printed in triplicate on the microarray by PE SpotArray72, UV crosslinked at 3000 mJ, and stored at 4°C.

### RNAi

RNAi was carried out by feeding worms with *E. coli *HT115 carrying plasmid L4440, expressing a dsRNA fragment of the targeted gene, using *E. coli *HT115 carrying a plasmid without insert as negative control. Plasmid L4440 (Addgene) contains two T7 promoters in opposite orientation at each side of the MCS thereby yielding dsRNA when transformed into bacterial strains expressing T7 polymerase. Plasmids targeting specific genes were constructed by inserting a 1–2 kb genomic PCR fragment (using primers pairs from Wormbase [55] of the targeted gene sequences, see Additional file 3) into plasmid L4440. The resulting construct was transformed into HT115 (an RNase III-deficient strain of *E. coli *with an isopropyl-β-D-thiogalactopyranoside (IPTG)-inducible T7 polymerase [56]) using standard CaCl_2 _transformation protocols and plated on 100 ug/ml ampiciline and 15 ug/ml tetracycline containing LB-agar plates and incubated at 37°C overnight. Selected colonies were minipreped (Promega) and the DNA subjected to restriction analysis. The bacterial cells were applied onto NGM plates [57] supplemented with 100 ug/ml ampicilline, 15 ug/ml tetracycline and 1 mM IPTG and incubated at room temperature for 2–3 days before adding the worms to the plates. RNAi phenotypes were observed after 24–72 hours. As positive controls for the RNAi we chose genes that produce distinct phenotypes; gpb-1, for which mutants are embryonic lethal, and unc-22, which results in a post-embryonic uncoordinated movements phenotype (Unc) [58,59]. Under optimised conditions, feeding the worms for 72 hours with plasmid target gpb-1 and unc-22, 100% dead embryos and 98% worms with uncoordinated movements [37] were produced, respectively. As an additional test, we fed worms expressing green fluorescent protein (GFP) with bacteria expressing double-stranded RNA (dsRNA) homologous to the gfp gene. Reduction in GFP fluorescence in the worms was observed by visual inspection under the microscope.

### Probes for Northern blotting

Digoxigenin-labeled RNA probes to dected depletion of mRNAs and snoRNAs on Northern blots were produced by in vitro transcription. *In vitro *transcriptions reactions were set up with 1 mM each of ATP, CTP and GTP, 0.65 mM UTP, and 0.35 mM Digoxigenin-11-UTP (Roche) in a 10 μl reaction volume, using an enzymatically digested plasmid with the relevant insert as template. The *in vitro *transcription reactions were incubated with T7 transcription polymerase (Invitrogen) at 37°C overnight and the reaction products were purified with Trizol (Invitrogen).

### Northern blotting

Northern blotting was carried out to confirm the depletion of mRNAs and snoRNAs. 1 μg of total RNA was denatured for 5 minutes at 70°C and loaded onto denaturing 6% polyacrylamide gels containing 7 M Urea. After electrophoresis for about 22 min at 250 V, RNA was transferred onto positively charged nylon membranes (Hybond-N+, Amersham Biosciences, UK. Cat. No. RPN303B). After brief washing using 2 × SSC, the transferred blots were cross-linked to the membrane under short-wave UV light. After prehybridization at 68°C for 1 hour in Ultrahyb Ultrasensitive Hybridizaion Buffer (Ambion), the blots were subjected to hybridization with Digoxigenin-labeled RNA probes overnight at 68°C. Then the membranes were washed as follows: twice for 5 minutes at room temperature in 2 × SSC containing 0.1% SDS, 15 minutes at 68°C in 2 × SSC containing 0.1% SDS, 10 minutes at room temperature in 1 × Washing Buffer (DIG Wash and Blocking Buffer Set, Roche). After the above mentioned steps, the membranes were blocked for 30 min at room temperature in 1 × Blocking Buffer (DIG Wash and Blocking Buffer Set, Roche), then incubated with anti-Digoxigenin AP Fab fragments (Roche) diluted (1/10000) in 1 × Blocking Buffer at room temperature for 30 min. After that, the membranes were washed twice (15 minutes each time) at room temperature in 1 × Washing Buffer, and then equilibrated in 1 × detection buffer (DIG Wash and Blocking Buffer Set, Roche) at room temperature for 3 minutes, followed by incubation with several drops of CDP-star (Roche) at room temperature for 30 minutes. Chemiluminescent signals were recorded with a ChemiCapt 3000 imaging system (Vilber).

### Microarray analysis

RNAi against protein components of snoRNPs was carried out as described above. After feeding stage L1 worms for 72 hours, total RNA was isolated by the Trizol (Invitrogen) protocol from both targeted and control worms. The total RNA was dephosphorylated with calf intestine alkaline phosphatase (Fermentas), and ligated to a 21 nt adapter oligonucleotide with T4 RNA ligase (Fermentas). The ligated ncRNAs were reverse transcribed using an oligonucleotide complementary to the 21 nt adapter, while the mRNA was reverse transcribed using an oligodT_12–18 _primer. The complementary DNA (cDNAs) from worms treated with RNAi was labeled with Cy5 (RNA from control worms was labeled with Cy3) using the Ambion Amino Allyl cDNA labeling kit. The microarrays were prehybridized at 50°C for 2 h and hybridized at 42°C for 14–16 h. Microarrays were scanned using Genepix 4000B scanner, and raw data were obtained and quantified with GenePix software.

### Computational methods

The raw data was processed using the MIDAS (TIGR TM4) software. Background was subtracted from the median pixel intensity values for Cy3 and Cy5, and data points were removed if intensities did not exceed 2-fold of background levels for both Cy3 and Cy5. Total intensity normalization and LocFit normalization were applied with housekeeping genes as controls. The MIDAS in-slide replicate analysis was applied to merge replicates of each gene. The relative expression levels (i.e. Cy5/Cy3 ratios) were log-transformed (base 2), and TMEV (TIGR TM4 software [60]) was used for hierarchical clustering. A Z-score was calculated for each gene under each condition (see Additional file 4). Genes with both Z-score and sample/reference ratio exceeding (or equal to) +2 were identified as significant. A two tailed student t-test was performed by comparison of ncRNAs in each functional class with all the 137 ncRNAs.

## Authors' contributions

MNA and HH performed experiments of RNAi and ncRNA microarray and interpreted the results. MNA and GS drafted the manuscript. RC and GS directed the design of the study. All the authors read and approved the final manuscript.

## Supplementary Material

Additional file 1 Northern and microarray figures. The data provided shows the expression levels of ncRNAs and proteins. Click here for file

Additional file 2 Secondary structures of unclassified ncRNAs. The data provided shows the secondary structures of six unclassified ncRNAs. Click here for file

Additional file 3 Primers. The data provided shows the primers used for RNAi experiments. Click here for file

Additional file 4 Z-score. The data provided described the definition of Z-score and the Z-score for all ncRNAs after depletion of C/D and H/ACA snoRNPs. Click here for file

Additional file 5 Clusters. The data provided shows the distribution of ncRNAs in seven clusters. Click here for file
