# Supplementary material for: Microarray analysis of ncRNA expression patterns in Caenorhabditis elegans after RNAi against snoRNA associated proteins
Source: BMC Genomics. 2008 Jun 11;9:278. doi: 10.1186/1471-2164-9-278 (PMC2442092; doi:10.1186/1471-2164-9-278)
Supplement: Additional file 1 — Northern and microarray figures. The data provided shows the expression levels of ncRNAs and proteins. [file 1471-2164-9-278-S1.pdf]

Northern and microarray Figures

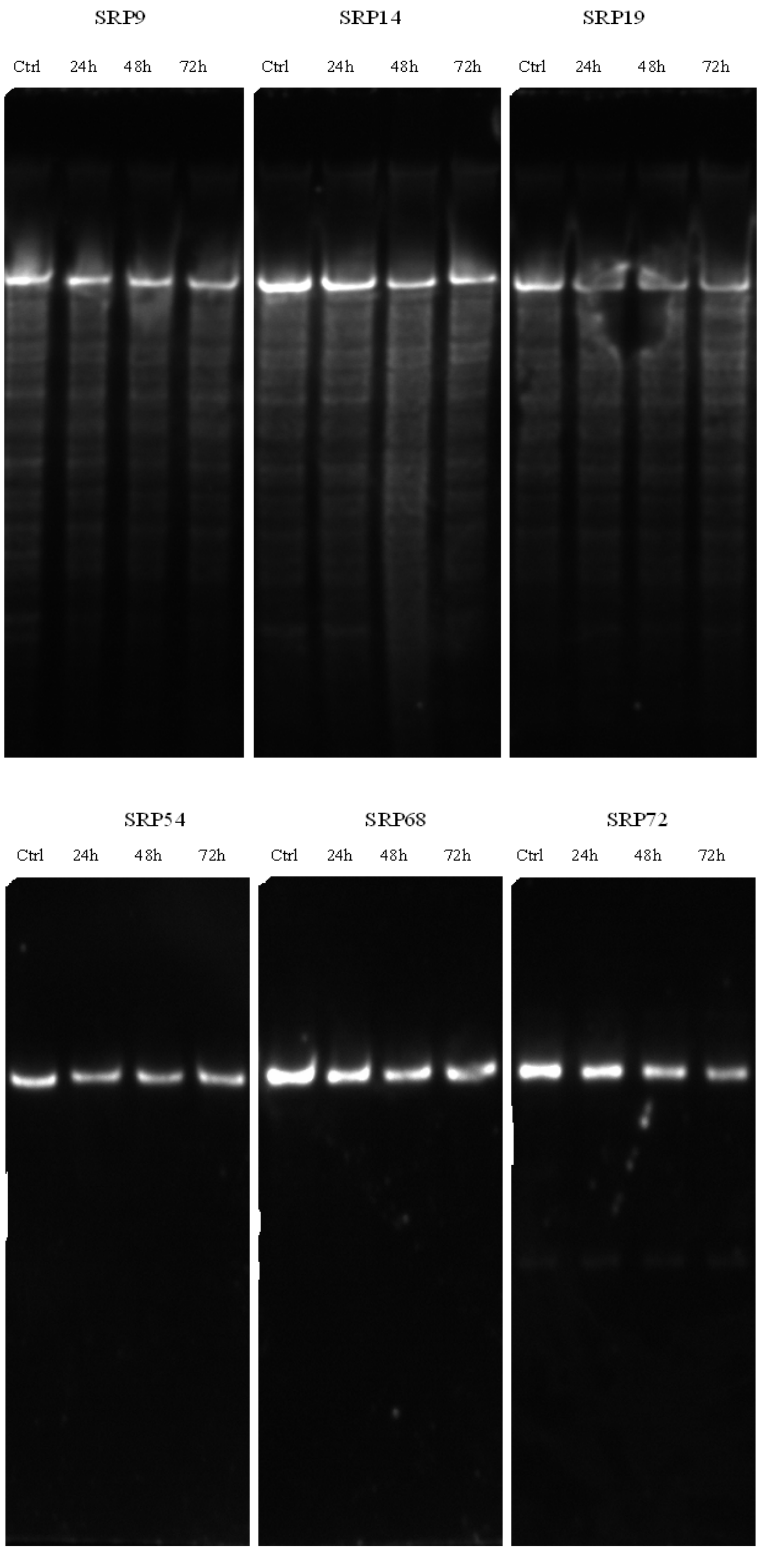

Figure 1. Effects of RNAi against SRP component proteins on SRP RNA expression levels. Northern blots of SRP RNA CeN107-1 after RNAi against SRP component proteins SRP9, SRP14, SRP19, SRP54, SRP68 and SRP72.

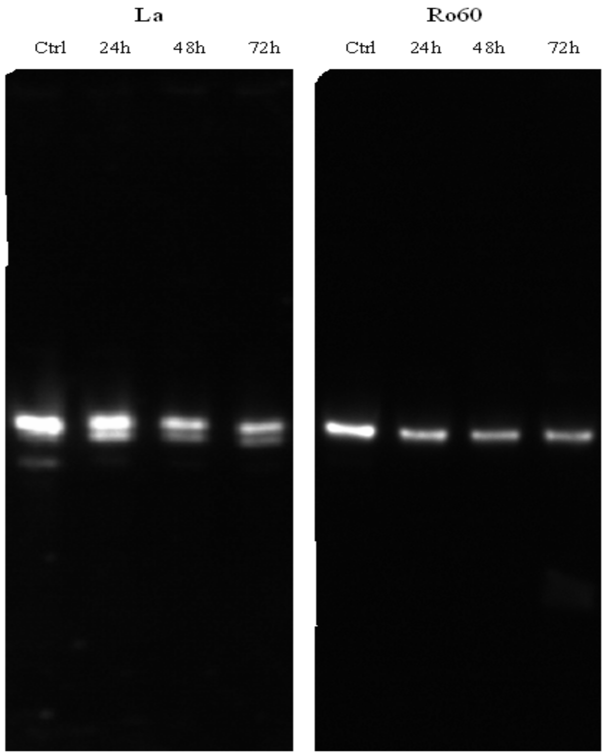

Figure 2. Effects of RNAi against Y RNP component proteins on Y RNA expression levels. Northern blots of Y RNA CeN9 after RNAi against Y RNP component proteins La and Ro60.

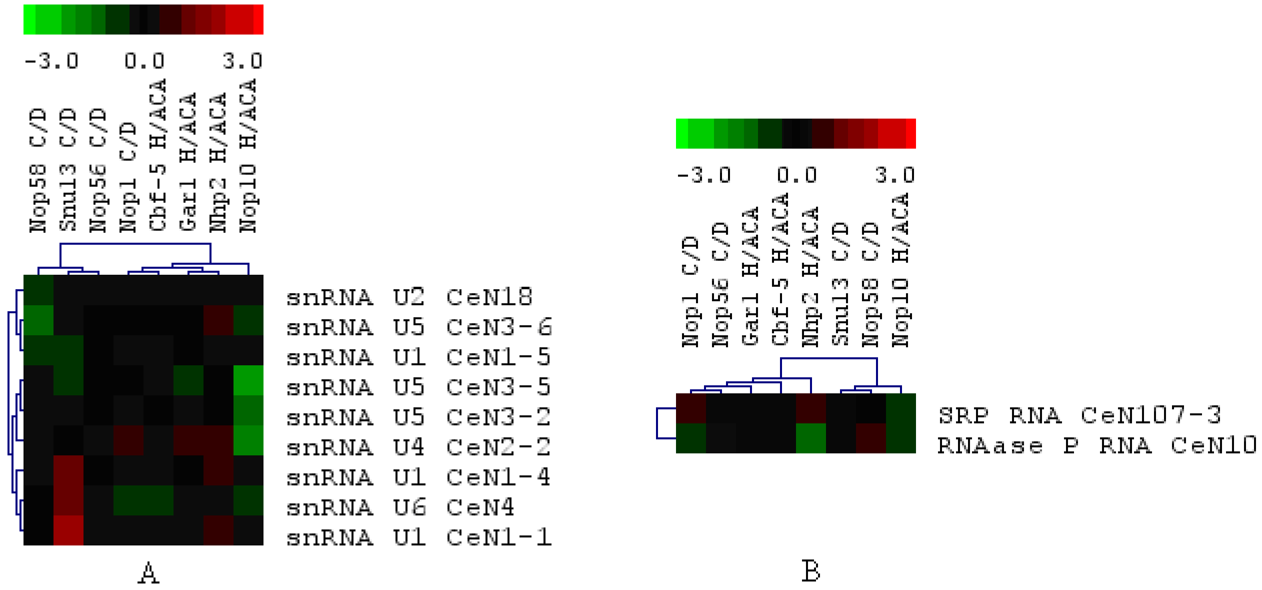

Figure 3. Depletion of C/D and H/ACA snoRNPs has almost negligible effect on A) snRNAs and B) SRP RNA and RNase P RNA.

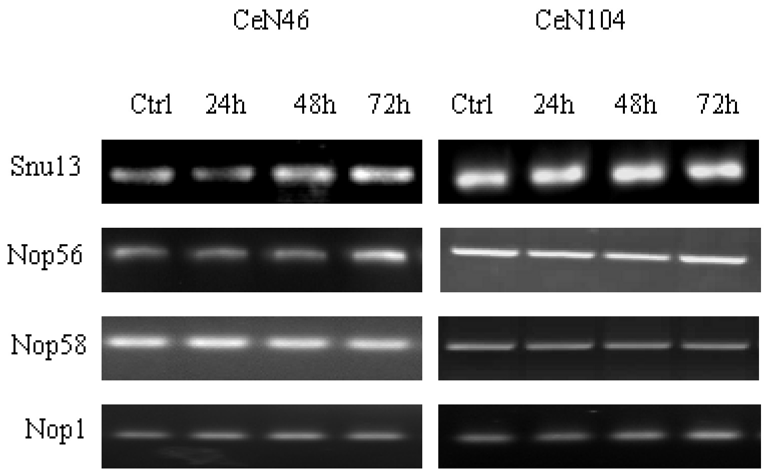

Figure 4. Effects of RNAi against the proteins Snu13, Nop56, Nop58 and Nop1 on the stability of H/ACA box snoRNA CeN46 and CeN104. The figure shows Northern blots of H/ACA box snoRNA CeN46 and CeN104 after RNAi against C/D box snoRNP proteins Snu13, Nop56, Nop58 and Nop1.

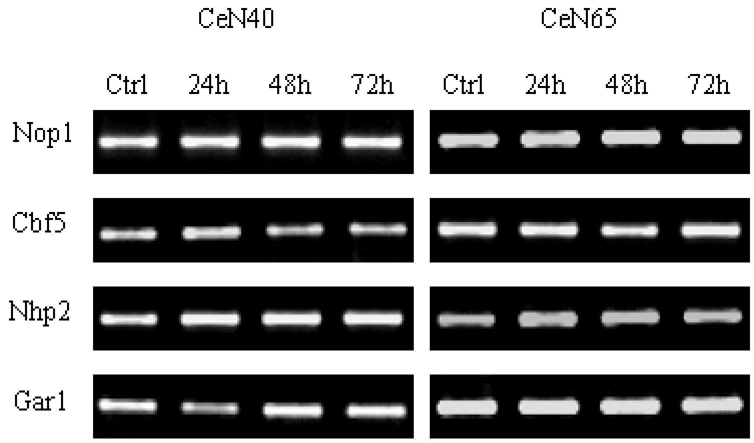

Figure 5. Effects of RNAi against the proteins Nop10, Cbf5, Nhp2 and Gar1 on the stability of C/D box snoRNA CeN40 and CeN65. The figure shows Northern blots of C/D box snoRNA CeN40 and CeN65 after RNAi against H/ACA box snoRNP proteins Nop10, Cbf5, Nhp2 and Gar1.

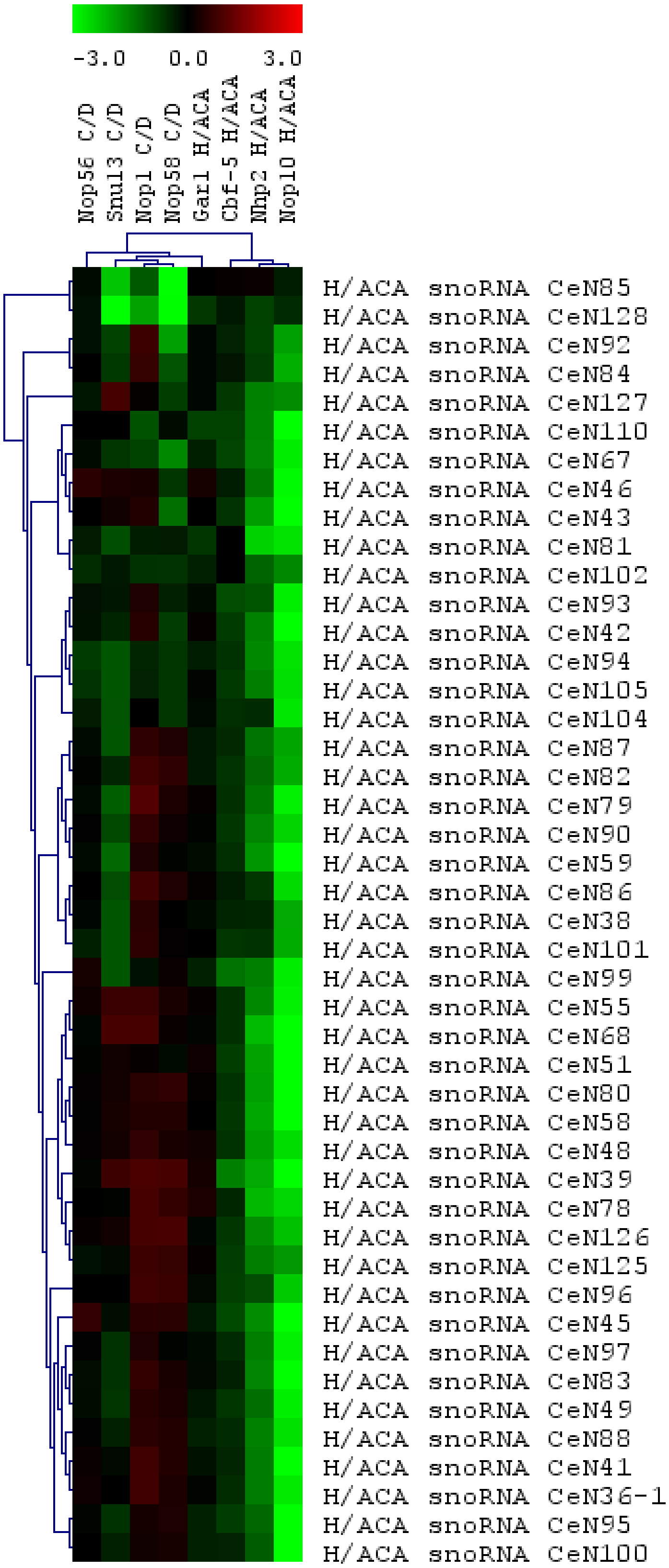

Figure 6. Expression profile of all H/ACA snoRNAs after depletion of C/D and H/ACA snoRNPs.

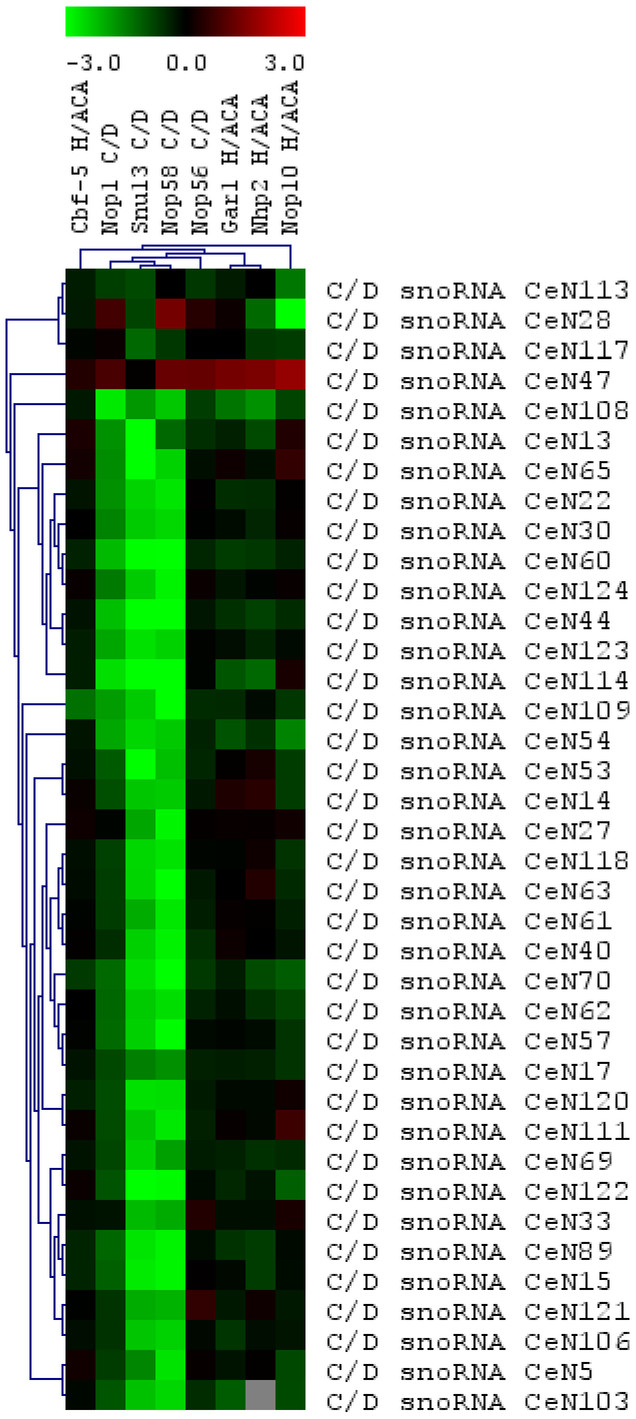

Figure 7. Expression profile of all C/D snoRNAs after depletion of C/D and H/ACA snoRNPs.

.

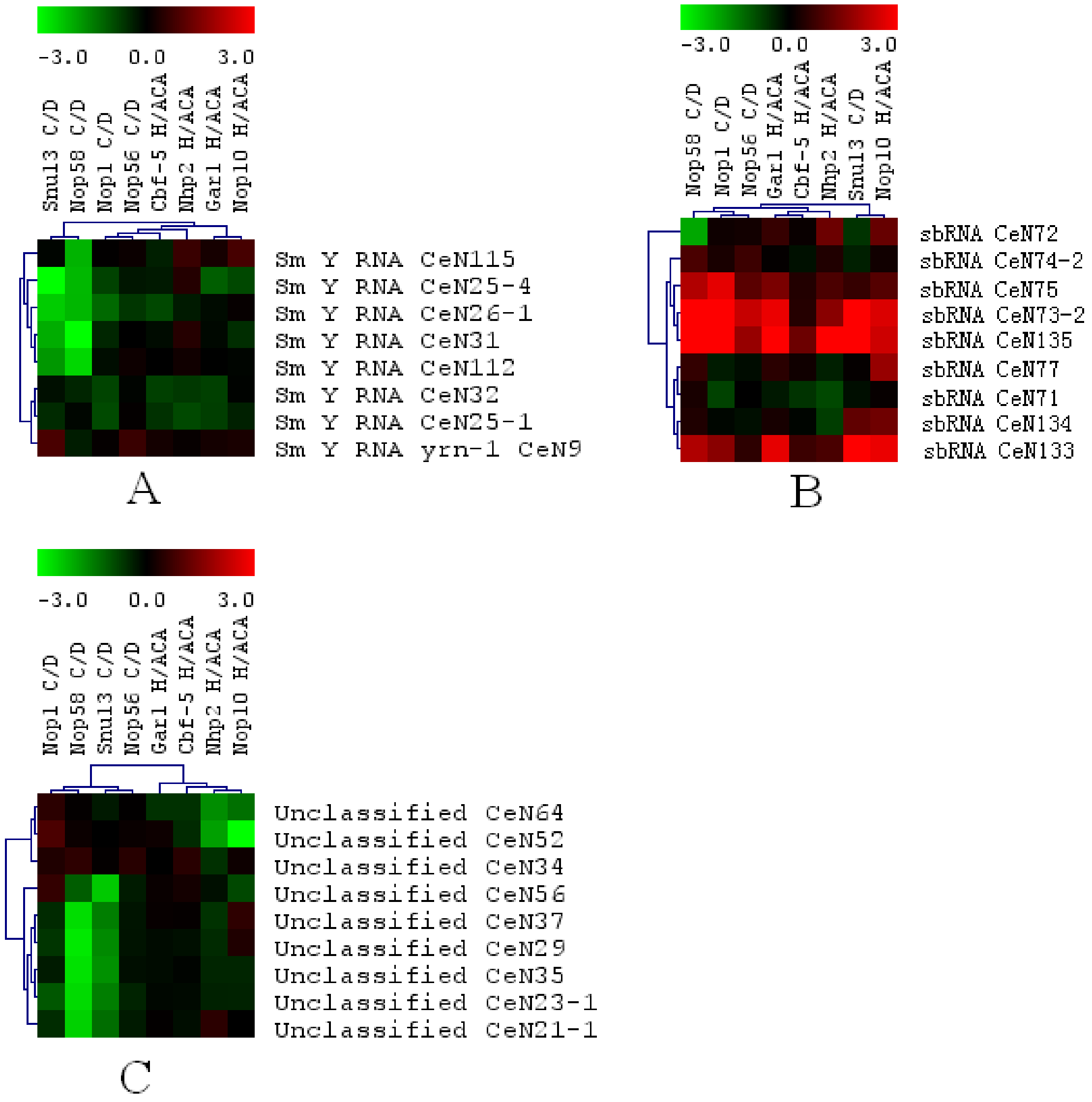

Figure 8. Expression profile of A) Sm Y RNAs B) sbRNAs. C) Unclassified ncRNAs, after depletion of C/D and H/ACA snoRNPs.
