# Supplementary material for: Microarray analysis of ncRNA expression patterns in Caenorhabditis elegans after RNAi against snoRNA associated proteins
Source: BMC Genomics. 2008 Jun 11;9:278. doi: 10.1186/1471-2164-9-278 (PMC2442092; doi:10.1186/1471-2164-9-278)
Supplement: Additional file 2 — Secondary structures of unclassified ncRNAs. The data provided shows the secondary structures of six unclassified ncRNAs. [file 1471-2164-9-278-S2.pdf]

Secondary structures of unclassified ncRNAs

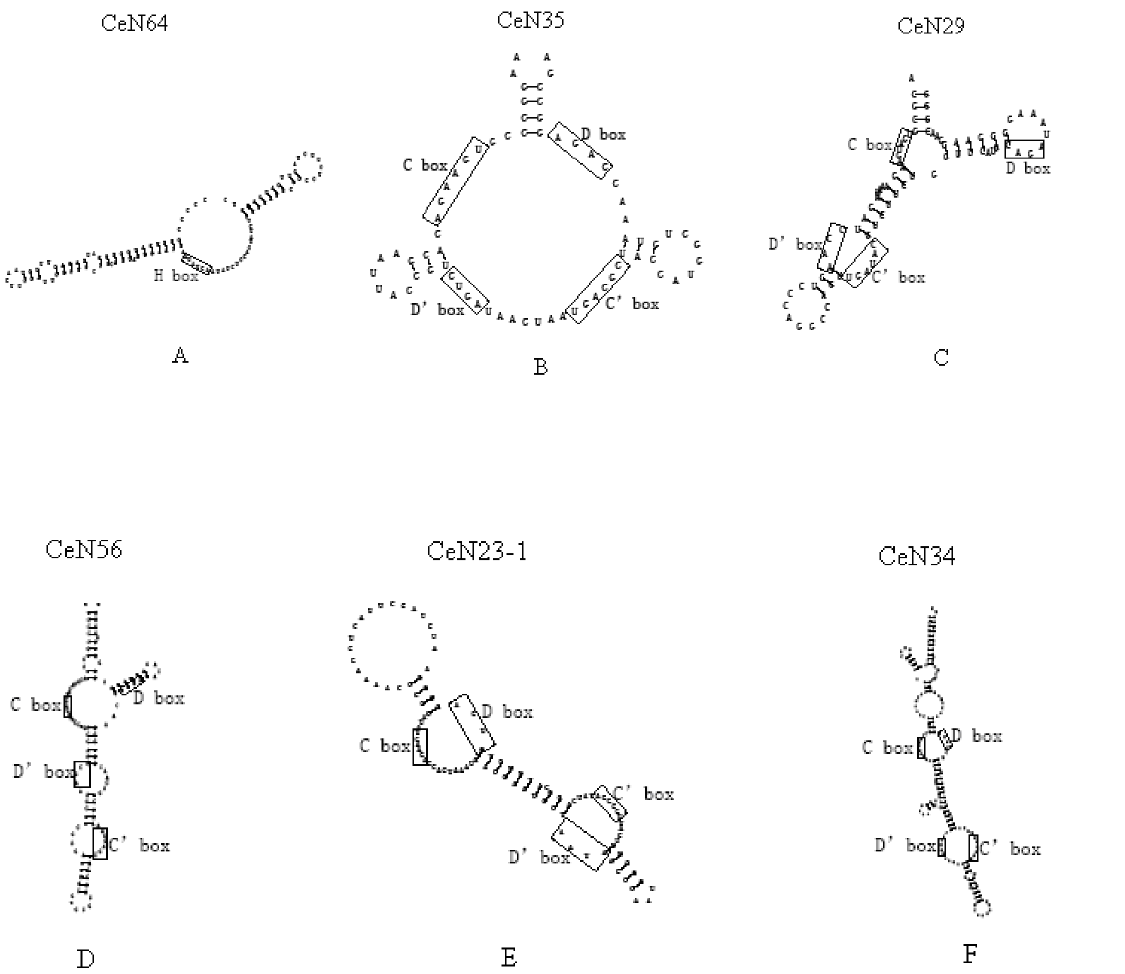

Figure 1. Secondary structures of unclassified ncRNAs A) CeN64 B) CeN35 and C) CeN29 D) CeN56 E) CeN23-1 and F) CeN34. Secondary structures show that CeN64 belongs to H/ACA class due to presence of H box and all other ncRNAs fall in C/D box snoRNA classification due to presence of specific C and D box sequence motifs.
