# Supplementary material for: Microarray analysis of ncRNA expression patterns in Caenorhabditis elegans after RNAi against snoRNA associated proteins
Source: BMC Genomics. 2008 Jun 11;9:278. doi: 10.1186/1471-2164-9-278 (PMC2442092; doi:10.1186/1471-2164-9-278)
Supplement: Additional file 3 — Primers. The data provided shows the primers used for RNAi experiments. [file 1471-2164-9-278-S3.pdf]

Primers

Following primer pairs were used from worm base primer pairs in the amplification of the snoRNA associated proteins. SacI site was added at 5’ end of all the forward primers and PaeI site was added at 5’ end of all reverse primers for Snu13, Nop1, Cbf5, Nop10 and Gar1. BglII at 5’ end of forward primers and XhoI at 5’ end of reverse primers were added for Nop58 and Gar1 while BglII and EcoRI was added repectively at 5’ end of forward and reverse primers respectively for Nop56 for cloning purpose. GC nucleotides were added before the enzymes sites at 5’ ends of both primers. GC clamp and enzyme sites are shown in bold.

Primers for C/D snoRNPs

**Snu13:**	Forward	5’-**GCGAGCTC**ATGTATTTGCAATTTGGGACAAC-3’
	Reverse	5’-**GCGCATGC**CTCTTTTCTTTTGTTTCTCCCGT-3’
**Nop58:**	Forward	5’-**GCAGATCT**GATTCACTTTTCTCACGATCCAC-3’
	Reverse	5’-**GCCTCGAG**ACCCTGTAAACCTGACAGAATCA-3’
**Nop56:**	Forward	5’-**GCAGATCT**GCACCAACTTGTTCTCCGAT-3’
	Reverse	5’-**GCGAATTC**GAGCTTGCTTCGTTTGATCC-3’
**Nop1:**	Forward	5’-**GCGAGCTC**CTCATACGGCTCCAGGGTTA-3’
	Reverse	5’-**GCGCATGC**TGTTCGCCATCAAAACGATA-3’

Primers for H/ACA box snoRNPs:

**Cbf5p:**	Reverse	5’-**GCGAGCTC**AAGCTCCACGAGGAAGTTGA-3’
	Forward	5’-**GCGCATGC**GTCGACACTGCCTGGAAACT-3’
**Nop10:**	Forward	5’-**GCGAGCTC**AATTCAACAAAACGAATTTCCCT-3’
	Reverse	5’-**GCGCATGC**TTGGAAATTACCGAGAAAGGATT-3’
**Nh2p:**	Reverse	5’-**GCAGATCT**AATTCAACAAAACGAATTTCCCT-3’
	Forward	5’-**GCCTCGAG**TTGGAAATTACCGAGAAAGGATT-3’
**Gar1:**	Reverse	5’-**GCGAGCTC**ATTCTTCGAAATAGGCGAAAATC-3’
	Forward	5’-**GCGCATGC**GCAGATGAGAAAAGGAAAACAAA-3’
