# Supplementary material for: Microarray analysis of ncRNA expression patterns in Caenorhabditis elegans after RNAi against snoRNA associated proteins
Source: BMC Genomics. 2008 Jun 11;9:278. doi: 10.1186/1471-2164-9-278 (PMC2442092; doi:10.1186/1471-2164-9-278)
Supplement: Additional file 4 — Z-score. The data provided described the definition of Z-score and the Z-score for all ncRNAs after depletion of C/D and H/ACA snoRNPs. [file 1471-2164-9-278-S4.pdf]

Z-score

Z score was calculated as follows:
Standard Deviation (SD) and Coefficient of Variation (CV) of the expression levels, and mean expression level (Em) of each ncRNA was calculated, CVmedian being the median of all CVs.

SD = sqrt(Σ(Ei–Em)$_2$)

(Ei being the expression level at the ith condition (i is from 1 to 9)).

CV = SD / E

A Z score was calculated as follows:

Zi = (Ei–Em)/MAX{SD, Em * CV median }

If | Zi $\geqslant$ 2|, and the sample/ reference ratio was greater than 2 or less than 0.5, Ei was considered as significantly over- (Zi $\geqslant$ 2) or under- (Zi $\leqslant$ -2) expressed.

Table 1. Z-score for all ncRNAs after depletion of C/D and H/ACA snoRNPs.

**Name_function**	**Z -score**							
	Nop10	Nhp2	Cbf5	Gar1	Nop58	Nop56	Snu13	Nop1
ceN64_un	2.88	2.20	1.68	1.44	-0.02	-0.23	0.09	-0.26
ceN56_un	0.01	0.04	0.00	-0.23	0.39	0.58	1.58	-0.33
ceN52_un	2.37	2.26	1.00	-0.33	-0.03	-0.28	0.00	-0.42
ceN37_un	0.24	0.24	-0.19	-0.20	1.75	0.80	0.68	0.40
ceN35_un	0.24	0.33	0.28	0.28	1.84	0.64	0.86	0.23
ceN34_un	0.19	0.39	-0.18	-0.02	-0.11	-0.01	-0.02	-0.24
ceN29_un	0.56	1.16	0.39	0.29	1.98	0.91	0.79	0.59
ceN23-1_un	0.31	0.91	0.56	0.18	1.71	0.95	0.68	0.95
ceN21-1_un	0.26	-0.16	0.39	-0.11	1.55	0.68	0.55	0.49
ceN107-3_SRP RNA	0.47	-0.29	0.20	-0.24	0.00	-0.11	0.08	-0.37
ceN4_snRNA U6	-0.1	0.26	0.35	0.57	0.00	-0.23	-0.20	0.41
ceN3-6_snRNA U5	-0.35	-0.13	1.17	0.03	0.40	-0.28	0.06	0.05
ceN3-5_snRNA U5	-0.03	0.39	0.54	1.12	0.04	-0.08	0.14	-0.02
ceN3-2_snRNA U5	-0.11	0.03	-0.13	0.45	0.04	-0.11	0.05	-0.08
ceN2-2_snRNA U4	-0.16	-0.38	0.21	-0.76	-0.07	-0.35	-0.02	-0.22
ceN18_snRNA U2	-0.44	-0.23	-0.28	-0.48	0.15	-0.30	-0.03	-0.16
ceN1-5_snRNA U1	-0.31	-0.09	-0.10	-0.11	0.21	0.16	0.11	0.02
ceN1-4_snRNA U1	-0.29	-0.44	-0.38	-0.18	0.05	-0.24	-0.18	-0.01
ceN1-1_snRNA U1	-0.09	-0.60	-0.32	-0.45	0.00	-0.35	-0.28	0.00
ceN8-1_snRNA sls-2	0.06	0.49	0.63	-0.01	0.94	0.16	0.38	-0.06
ceN7_snRNA sls-2	-0.10	0.67	0.55	1.12	0.23	-0.20	0.64	-0.16
ceN6_snRNA sls-2	0.42	0.43	0.27	0.32	0.08	-0.19	-0.04	-0.23
ceN20_snRNA sls-2	0.01	0.13	0.54	0.71	0.78	-0.20	0.09	0.06
ceN19_snRNA sls-2	0.43	0.45	-0.13	-0.09	0.15	-0.09	0.07	-0.04
ceN16-4_snRNA sls-2	0.18	1.88	1.59	1.01	0.01	0.09	0.61	-0.10

ceN16-3_snRNA sls-2	0.03	0.74	1.18	0.49	-0.02	-0.03	0.42	-0.24
ceN16-1_snRNA sls-2	0.10	1.13	1.35	0.64	-0.01	0.05	0.90	-0.14
ceN12_snRNA sls-2	-0.19	0.07	0.63	1.19	0.53	-0.21	0.25	-0.08
ceN116_snRNA SL1	0.63	0.94	0.86	0.36	0.24	0.24	0.70	0.30
ceN86_snoRNA H/ACA	1.32	0.75	0.77	-0.16	-0.08	-0.10	0.33	-0.37
ceN55_snoRNA H/ACA	2.25	2.13	1.27	-0.18	-0.07	-0.12	-0.14	-0.33
ceN46_snoRNA H/ACA	1.50	1.04	-0.06	-0.51	0.19	-0.26	-0.08	-0.15
ceN110_snoRNA H/ACA	1.29	1.94	2.04	2.02	0.04	0.44	0.38	0.73
ceN99_snoRNA H/ACA	1.28	1.72	2.02	0.91	-0.03	0.07	0.55	0.17
ceN97_snoRNA H/ACA	1.76	2.20	1.17	0.25	0.01	-0.01	0.59	-0.22
ceN96_snoRNA H/ACA	1.74	1.31	1.40	0.22	-0.13	0.04	0.67	-0.33
ceN95_snoRNA H/ACA	1.43	2.07	2.07	0.90	-0.08	0.12	0.00	-0.16
ceN94_snoRNA H/ACA	1.42	2.69	1.29	0.76	0.19	1.24	0.19	0.30
ceN93_snoRNA H/ACA	1.71	1.27	2.31	0.29	0.10	0.40	0.00	-0.21
ceN92_snoRNA H/ACA	1.61	1.05	1.25	0.18	0.94	-0.03	0.27	-0.34
ceN90_snoRNA H/ACA	1.67	1.72	2.02	0.10	-0.04	0.05	0.38	-0.36
ceN88_snoRNA H/ACA	1.50	1.95	1.44	0.88	-0.09	-0.02	0.27	-0.27
ceN87_snoRNA H/ACA	1.66	1.92	1.57	0.60	-0.08	0.09	0.38	-0.29
ceN85_snoRNA H/ACA	0.05	-0.26	0.23	-0.03	2.43	0.42	0.76	1.14
ceN84_snoRNA H/ACA	0.84	1.08	0.66	0.19	0.34	0.02	0.00	-0.33
ceN83_snoRNA H/ACA	1.97	1.77	0.88	0.20	-0.07	0.06	0.36	-0.31
ceN82_snoRNA H/ACA	2.09	1.27	0.96	0.60	-0.11	0.00	0.13	-0.37
ceN81_snoRNA H/ACA	1.69	3.99	1.14	1.58	0.09	0.45	0.34	0.29
ceN80_snoRNA H/ACA	2.54	2.64	2.32	-0.10	-0.11	0.06	-0.06	-0.28
ceN79_snoRNA H/ACA	2.09	1.59	1.70	-0.18	-0.07	-0.02	0.00	-0.46
ceN78_snoRNA H/ACA	2.43	2.89	0.93	-0.61	-0.12	-0.03	0.01	-0.39
ceN68_snoRNA H/ACA	2.73	2.76	2.45	0.13	-0.03	0.23	-0.17	-0.34
ceN67_snoRNA H/ACA	1.93	2.25	2.22	0.82	0.71	0.50	0.70	0.71
ceN59_snoRNA H/ACA	2.73	2.55	1.73	0.32	0.01	0.21	0.00	-0.19
ceN58_snoRNA H/ACA	3.00	2.12	1.01	-0.01	-0.09	0.01	-0.07	-0.18
ceN51_snoRNA H/ACA	2.80	2.42	1.54	-0.33	0.03	0.03	-0.05	-0.07
ceN49_snoRNA H/ACA	1.90	2.07	2.14	0.56	-0.07	0.13	0.00	-0.28
ceN48_snoRNA H/ACA	2.09	2.65	1.16	-0.38	-0.07	-0.03	-0.06	-0.32
ceN45_snoRNA H/ACA	1.69	2.47	3.22	0.62	-0.10	-0.04	0.04	-0.29
ceN43_snoRNA H/ACA	1.84	2.05	0.99	-0.05	0.51	0.11	-0.05	-0.19
ceN42_snoRNA H/ACA	1.58	1.74	1.69	-0.18	0.23	0.21	0.13	-0.28
ceN41_snoRNA H/ACA	2.07	1.15	0.86	0.44	-0.09	-0.11	0.03	-0.41
ceN39_snoRNA H/ACA	2.63	2.34	1.58	-0.48	-0.15	0.25	-0.15	-0.43
ceN38_snoRNA H/ACA	1.10	0.91	1.03	0.29	0.00	0.14	0.00	-0.28
ceN36-1_snoRNA H/ACA	1.46	1.29	1.35	0.13	-0.07	-0.15	0.00	-0.34
ceN128_snoRNA H/ACA	-0.02	1.48	1.26	1.70	2.41	1.14	4.71	2.54
ceN127_snoRNA H/ACA	3.46	2.17	1.93	0.17	0.23	0.65	0.59	-0.01

ceN126_snoRNA H/ACA	2.75	2.34	1.42	0.18	-0.16	-0.02	-0.05	-0.39
ceN125_snoRNA H/ACA	2.91	1.99	1.77	-0.20	-0.12	0.12	0.03	-0.37
ceN105_snoRNA H/ACA	2.32	1.65	3.70	0.12	0.60	0.56	0.00	0.21
ceN104_snoRNA H/ACA	1.82	0.47	1.37	0.24	0.56	0.29	0.37	-0.01
ceN102_snoRNA H/ACA	1.75	1.36	-0.15	0.92	0.18	0.94	0.08	0.64
ceN101_snoRNA H/ACA	1.54	0.74	1.37	-0.07	-0.01	0.74	0.00	-0.28
ceN100_snoRNA H/ACA	0.95	1.51	1.38	0.92	-0.06	0.08	0.00	-0.16
ceN44_snoRNA C/D possible	-0.14	0.71	0.75	1.38	2.80	1.42	4.22	3.45
ceN113_snoRNA C/D possible	0.16	-0.14	1.86	0.71	0.00	0.94	0.31	-0.05
ceN118_snoRNA C/D	0.25	0.03	0.65	0.11	1.87	0.40	1.80	0.54
ceN89_snoRNA C/D	0.02	1.25	2.04	1.45	2.20	0.77	1.45	1.19
ceN70_snoRNA C/D	-0.12	0.48	0.85	0.77	2.86	2.06	1.14	1.36
ceN69_snoRNA C/D	0.24	1.14	0.62	0.91	0.93	0.71	1.74	0.71
ceN65_snoRNA C/D	-0.25	0.67	0.18	-0.36	1.58	1.03	4.68	1.90
ceN63_snoRNA C/D	-0.03	-0.15	0.25	0.02	2.49	1.28	1.77	0.52
ceN62_snoRNA C/D	-0.10	0.50	1.10	0.36	1.77	1.51	1.28	1.22
ceN61_snoRNA C/D	0.12	0.23	0.31	-0.20	1.93	1.25	1.03	0.53
ceN60_snoRNA C/D	0.41	1.09	2.18	1.84	2.87	2.58	2.12	3.23
ceN57_snoRNA C/D	0.12	-0.08	0.33	0.13	2.37	0.50	1.13	1.22
ceN54_snoRNA C/D	0.68	1.11	1.69	2.83	1.43	3.04	1.79	2.63
ceN53_snoRNA C/D	0.53	-0.06	0.78	-0.10	1.29	1.00	2.64	0.29
ceN5_snoRNA C/D	-0.07	-0.24	-0.47	0.51	1.86	0.14	0.70	0.55
ceN47_snoRNA C/D	-0.40	-0.73	-0.84	-1.78	-0.20	-0.64	-0.01	-0.44
ceN40_snoRNA C/D	-0.01	0.05	0.06	-0.31	2.15	1.44	1.69	0.14
ceN33_snoRNA C/D	0.42	0.71	0.38	0.36	1.05	0.00	1.36	0.07
ceN30_snoRNA C/D	0.39	0.23	0.06	0.28	1.68	0.39	0.65	1.73
ceN28_snoRNA C/D	1.33	1.38	0.42	-0.30	-0.21	-0.38	0.28	-0.58
ceN27_snoRNA C/D	-0.28	-0.24	-0.35	-0.21	2.23	0.02	0.81	0.07
ceN22_snoRNA C/D	0.64	1.17	0.81	1.31	1.91	0.50	1.74	2.00
ceN17_snoRNA C/D	0.69	0.69	0.84	0.78	0.69	0.93	0.41	0.57
ceN15_snoRNA C/D	0.02	0.35	0.55	0.21	2.22	0.62	0.55	1.03
ceN14_snoRNA C/D	0.09	-0.08	0.30	-0.67	1.47	0.58	1.53	0.83
ceN13_snoRNA C/D	-0.32	1.63	0.49	0.87	0.47	2.60	3.26	2.05
ceN124_snoRNA C/D	0.38	-0.13	0.38	0.55	2.19	0.24	0.89	1.54
ceN123_snoRNA C/D	0.99	0.57	2.07	0.33	1.58	0.57	1.35	2.43
ceN122_snoRNA C/D	0.12	0.78	0.56	1.11	2.33	0.43	3.72	0.88
ceN121_snoRNA C/D	0.18	0.19	0.47	0.63	1.15	-0.23	1.17	0.22
ceN120_snoRNA C/D	0.65	0.20	0.59	0.22	1.78	0.72	0.63	0.28
ceN117_snoRNA C/D	0.91	0.69	0.37	-0.07	0.20	0.00	0.51	-0.09
ceN114_snoRNA C/D	-0.13	1.68	1.54	2.94	2.65	1.37	3.82	4.54
ceN111_snoRNA C/D	-0.05	-0.17	-0.08	-0.17	1.98	1.28	1.36	0.82

ceN109_snoRNA C/D	1.33	0.38	2.47	1.16	2.90	3.15	1.35	1.08
ceN108_snoRNA C/D	2.59	2.82	2.11	4.62	1.41	8.30	0.80	5.50
ceN106_snoRNA C/D	-0.19	0.19	1.23	1.52	1.56	0.46	1.16	0.31
ceN103_snoRNA C/D	0.23	0.52	1.49	3.23	1.65	0.89	1.45	0.87
ceN32_snlRNA	0.32	1.28	1.19	2.01	0.12	0.62	0.05	0.27
ceN31_snlRNA	-0.42	-0.03	0.54	0.22	2.57	-0.48	1.18	0.35
ceN26-1_snlRNA	0.87	0.56	0.72	0.28	1.20	1.55	0.71	0.86
ceN25-4_snlRNA	-0.49	0.82	2.15	3.36	1.21	0.46	3.96	0.62
ceN25-1_snlRNA	0.72	1.74	1.20	1.91	0.02	0.52	0.16	0.08
ceN115_snlRNA	0.04	-0.64	-0.21	-0.50	1.15	-0.42	0.02	-0.04
ceN112_snlRNA	-0.30	-0.17	-0.30	0.03	1.65	-0.25	0.93	0.11
ceN9_scRNA yrn-1	-0.38	-0.35	-0.39	-0.46	0.09	-0.55	-0.17	-0.05
ceN77_sbRNA	0.21	0.25	-1.13	-0.87	-0.12	0.46	-0.02	-0.29
ceN75_sbRNA	-0.43	-0.66	-1.32	-1.83	-0.26	-0.73	-0.14	-0.79
ceN74-2_sbRNA	0.28	-0.50	-0.39	-0.13	-0.16	-0.08	0.11	-0.26
ceN73-2_sbRNA	-0.64	-1.12	-1.75	-2.46	-0.31	-1.19	-0.35	-0.87
ceN72_sbRNA	-0.46	-0.60	-0.45	-1.05	1.01	-0.18	0.20	-0.15
ceN71_sbRNA	0.75	0.78	0.54	0.65	-0.06	0.64	0.05	0.58
ceN10_RNAase P RNA	-0.19	1.40	1.08	0.51	-0.11	0.53	0.05	0.65
CeN131_predicted	0.17	0.22	0.68	1.12	2.52	0.83	1.94	0.76
CeN142_predicted	-0.18	0.21	0.41	0.36	2.91	0.41	1.08	1.56
p2480_predicted	0.32	-0.13	0.63	-0.23	-0.05	0.18	0.21	1.28
CeN130_predicted	-0.23	0.30	0.38	0.75	2.44	0.50	1.46	1.00
CeN136_predicted	0.94	1.35	1.88	1.78	2.54	2.11	1.17	2.81
CeN132_predicted	0.24	0.77	1.07	1.29	2.67	1.80	1.31	1.96
CeN141_predicted	0.48	0.43	2.11	1.29	2.46	0.13	0.66	0.57
CeN134_sbRNA	0.95	0.30	-1.00	-0.52	-0.08	2.30	-0.21	0.08
CeN135_sbRNA	-1.02	-1.42	-3.07	-2.78	-0.33	-1.28	-0.35	-0.92
CeN133_sbRNA	-0.40	-0.94	-2.52	-2.44	-0.26	-0.53	-0.35	-0.68
