# Supplementary material for: Microarray analysis of ncRNA expression patterns in Caenorhabditis elegans after RNAi against snoRNA associated proteins
Source: BMC Genomics. 2008 Jun 11;9:278. doi: 10.1186/1471-2164-9-278 (PMC2442092; doi:10.1186/1471-2164-9-278)
Supplement: Additional file 5 — Clusters. The data provided shows the distribution of ncRNAs in seven clusters. [file 1471-2164-9-278-S5.pdf]

Distribution of ncRNAs in the seven clusters (See Figure 3 in the main text).

Cluster	Functional class						Probability (P)
	C/D box snoRNAs	H/ACA snoRNAs	Sm Y RNAs	snRNAs	sbRNAs	Other ncRNAs[a]	
1	0	0	0	**3**	0	1	$<10^{-22}$
2	3	0	1	**5**	1	1	$<10^{-20}$
3	**32**	2	4	0	1	11	$<10^{-20}$
4	1	**42**	0	3	0	1	$<10^{-30}$
5	2	1	0	**8**	0	2	$<10^{-19}$
6	0	0	0	0	**3**	0	$<10^{-13}$
7	0	0	2	0	**4**	3	$<10^{-11}$
All	38	45	7	19	9	19	

The expression data were hierarchically clustered using TMEV3.0 (TM4 software [1]), resulting in seven clusters with distinct expression patterns, generally overlapping with ncRNA functional groups. The dominating ncRNA class in each cluster is indicated in **bold**. "Probability" indicates the calculated likelihood of finding at least the observed number of "dominating ncRNAs" in each cluster (P= $C_n^m C_{137-n}^{x-m} / C_{137}^x$. "n" represents number of ncRNAs in each functional class, "m" represents the number of ncRNAs in the dominant functional class in each cluster, "x" represents the number of ncRNAs in each cluster).

[a] "Other ncRNAs" include SRP RNA, RNase P RNA and unclassified ncRNAs.

1. Saeed AI, Sharov V, White J, Li J, Liang W, Bhagabati N, Braisted J, Klapa M, Currier T, Thiagarajan M *et al*: **TM4: a free, open-source system for microarray data management and analysis**. *Biotechniques* 2003, **34**(2):374-378.
